# Biocatalytic degradation of environmental endocrine disruptor chlorobenzene via surfactant-optimized laccase-mediator system

**DOI:** 10.3389/fbioe.2024.1469029

**Published:** 2024-10-14

**Authors:** Dan Wang, Guifang Huang, Chunming Yu, Yawen Wang, Nawon Baek, Ruofei Zhu

**Affiliations:** ^1^ College of Textile and Clothing, Xinjiang University, Urumqi, China; ^2^ Xinjiang Key Laboratory of Intelligent and Green Textile, Xinjiang University, Urumqi, China; ^3^ Department of Clothing and Textiles, Kyungpook National University, Daegu, Republic of Korea; ^4^ Center for Beautiful Aging, Kyungpook National University, Daegu, Republic of Korea

**Keywords:** laccase, chlorobenzenes, degradation, estrogenic activity, molecular simulation

## Abstract

The emergence of environmental endocrine disruptor chlorobenzene (CB) in surface water and its potential environmental impacts have attracted serious global attention. It is still very difficult to achieve effective degradation of it by catalytic oxidation process under mild conditions. Here, an optimized method for degrading CB in aqueous solution using *Trametes versicolor* laccase and surfactant-assisted laccase-mediator (SALM) system was investigated. The use of a Tween 80 surfactant enhanced the solubility of CB and promoted its efficient degradation. Under favorable conditions, the SALM system yielded a degradation efficiency of 43.5% and a dechlorination efficiency of 41.55% for CB (25 mg/L) within 24 h. The possible degradation pathway of CB by this system was speculated by detecting the intermediates produced during the reaction. The outcome of the proliferation assays on MCF-7 human breast cancer cells demonstrated a reduction in the estrogenic activity of the CB solution following treatment with the SALM system. Furthermore, the influence of the quantity and positional variation of chlorine substituents on the degradation process was methodically investigated. Moreover, molecular analyses were employed to study the detailed interaction mechanism between laccase and CB, which revealed that the hydrophobic interaction contributed dominantly to binding process. These findings provide an efficient and environmentally friendly degradation system for the development of purification strategies for halogenated pollutants.

## 1 Introduction

Chlorobenzenes (CBs), categorized within halogenated aromatic hydrocarbons, are primarily employed as precursor compounds in the fabrication of various chemical entities, encompassing dyes, aromatics, pest control agents, and solvents for chemical manufacturing processes (Ahsan et al., 2023; Hu et al., 2022). These compounds have elicited significant concerns due to their persistent, bio-accumulative nature, and estrogenic effects, meriting their classification as endocrine disruptor chemicals (EDCs) (Jose and Philip, 2019). Extended exposure of humans to CBs is correlated with a range of negative health effects, such as adverse effects on the central nervous system, elicitation of dermatological hypersensitivities, mucosal irritations, and significant disturbances to endocrine, reproductive, and immunological systems. Acknowledging the significant health risks associated with CBs, the U.S. Environmental Protection Agency (EPA) has designated CBs as a priority pollutant and stipulated a maximum permissible concentration of 100 μg/L (Feng et al., 2023).

The mitigation of CBs in the environment can be addressed through a variety of techniques, including physical, advanced oxidation, and biological methods, as well as their integrative approaches. For instance, Khalaf and Rohani (2017) utilized self-synthesized zeolite X to adsorb CB from wastewater, achieving a removal efficiency of 97.2% under optimized conditions. While adsorption is widely applied in water treatment, it primarily concentrates pollutants on the adsorbent’s surface or interior, thus failing to achieve complete pollutant elimination (Wen et al., 2023). Several advanced oxidation processes (AOPs), such as electrochemical treatment, Fenton’s reaction, ultrasonic degradation, photocatalytic degradation, and microwave irradiation, have been explored for CBs degradation (Jafari et al., 2022; Luo et al., 2023; Ye et al., 2024; Zhang X. et al., 2022). Nonetheless, these conventional AOPs are often associated with potential drawbacks, including the risk of secondary pollution, incomplete mineralization of contaminants, and complex operational requirements. In contrast, the biological degradation of CB is increasingly recognized as a sustainable and ecologically benign remediation strategy, preserving the integrity of the ecosystem. Aerobic microorganisms and microbial enzymes have been extensively documented for their efficacy in CB degradation (Liu et al., 2024; Trueba-Santiso et al., 2022; Zhang X.-P. et al., 2022). However, the application of free enzymes in degrading CB remains relatively underexplored. The use of free enzymes for wastewater remediation holds promising potential due to their substrate-specific activity, rapid treatment cycles, robust stability, and inherent environmental compatibility.

Laccase (EC 1.10.3.2), a member of the multinuclear copper-containing oxidases family, is proficient in catalyzing the oxidative degradation of a wide array of substrates, including phenols, aromatic amines, and their derivatives (Sun et al., 2023). Additionally, the enzyme is also capable of oxidizing certain high-molecular-weight non-phenolic substrates when paired with an appropriate redox mediator (Wang et al., 2023; Zhou et al., 2024). The laccase-mediator system has found extensive applications in the breakdown of various environmental pollutants, ranging from synthetic dyes and personal care products to herbicides (Barrios-Estrada et al., 2018; Bilal et al., 2019; Khalid et al., 2022). Despite its broad utility, the use of laccase in the bioremediation of certain hydrophobic contaminants is impeded by a notable reduction in enzyme activity, often attributed to the interaction with oxidative byproducts of the contaminants, resulting in suboptimal degradation efficiency (Budeli et al., 2022). To address this limitation, the introduction of nonionic surfactant additives into the reaction milieu has been proposed. These surfactants enhance the solubility of hydrophobic pollutants and, owing to their neutral charge, minimally impact the structural integrity of laccase (Li et al., 2023; Yao et al., 2024). Nevertheless, comprehensive studies and detailed insights into the biodegradation of CB via laccase-mediator systems in conjunction with nonionic surfactant remain relatively unexplored. This gap underscores the need for further investigation into optimizing such biocatalytic systems for efficient and environmentally friendly remediation of CB and similar recalcitrant pollutants.

The aim of this work is to scrutinize the efficacy of the surfactant-assisted laccase-mediator (SALM) system in the degradation and detoxification of CB, thereby diminishing their detrimental ecological effects. The influence of the operational parameters on CB degradation and surfactant on the stability of laccase during the process were studied. The proficiency of the SALM system in dechlorinating CB was assessed via ion chromatography spectroscopy (ICS) analysis. Concurrently, the metabolites generated during the degradation were identified and analyzed to deduce plausible pathways of CB degradation. Estrogenic activity assays were also conducted to assess the detoxification potential of laccase for CB. Moreover, intricate interactions between laccase and CB were elucidated through molecular docking and molecular dynamics simulations. These comprehensive investigations provide insights into the potential of the SALM system for the bioremediation of wastewater laden with obstinate pollutants.

## 2 Materials and methods

### 2.1 Materials

Chlorobenzene (CB), 1, 2-Dichlorobenzene (1, 2-DCB), 1, 3-Dichlorobenzene (1, 3-DCB), 1, 4-Dichlorobenzene (1, 4-DCB), 2′2-azino-bis [3-ethylbenzothiazoline-6-sulphonic acid] (ABTS), 1-hydroxybenzotriazole (HBT), 2, 2, 6, 6-tetramethylpiperidine-1-oxyl (TEMPO), and Tween 80 were acquired from Aladdin (Shanghai, China). Laccase (EC.1.10.3.2) sourced from *Trametes versicolor* was procured from Sigma-Aldrich (Shanghai, China). N-hexane, acetonitrile, and methanol, all of HPLC grade, along with other analytical grade chemicals, were supplied by Sinopharm Chemical Reagent Co., Ltd. (Shanghai, China).

### 2.2 Determination of laccase activity

The catalytic activity of laccase was quantified through UV-Visible spectrophotometry, observing the kinetic transformation of 0.5 mM ABTS (ε_420_ = 36,000 M^−1^ × cm^−1^) to its oxidized form, ABTS^+^, at a wavelength of 420 nm within a 50 mM acetate buffer solution (pH 4.5) maintained at 30°C. The enzymatic activity was delineated in units, each corresponding to the quantity of laccase necessary to facilitate the oxidation of 1 µmol of ABTS per minute.

### 2.3 CBs degradation by laccase in an aqueous system

The degradation assays were conducted at a constant temperature of 30°C with magnetic stirring over a period of 24 h within Erlenmeyer flasks. These flasks each contained 20 mL of a reaction mixture. This mixture comprised 25 mg/L of CB, 50 mM acetate buffer maintaining a pH of 5.0, 1.0 U/mL of laccase, 0.16 mM of TEMPO, and 0.05% (v/v) of Tween 80. To ensure reproducibility and statistical reliability, all experimental runs were performed in triplicate. At predetermined intervals, aliquots of 0.5 mL were meticulously extracted to monitor the progression of the reaction. Subsequently, an equivalent volume of n-hexane was introduced to each aliquot, followed by vigorous vortex mixing for 5 min. This procedure enabled the segregation of reaction byproducts into discrete aqueous and organic phases via centrifugation at 3,000 rpm for a duration of 15 min. Subsequently, the organic layer was carefully isolated and filtered through a 0.22 μm pore-size organic membrane filter, rendering it suitable for ensuing analytical evaluations.

### 2.4 Influence of CBs structural configuration on enzymatic degradation efficacy

Four congeners of CBs (CB, 1, 2-DCB, 1, 3-DCB, 1, 4-DCB) were evaluated to ascertain the impact of chlorination pattern (number and positional distribution of -Cl groups) on their enzymatic degradation by the SALM system. Reaction mixtures, each comprising 25 mg/L of CB, 1, 2-DCB, 1, 3-DCB, or 1, 4-DCB, were subjected to incubation at 30°C for 24 h in the presence of 1.0 U/mL laccase, 0.16 mM TEMPO, and 0.1% Tween 80, individually.

### 2.5 Analytical procedure

The quantification of CBs in liquid samples was conducted using a Shimadzu LC-16 HPLC system, featuring an SPD-16 UV detection unit. Chromatographic separation was achieved with an SB-C_18_ reversed-phase column (5 μm, 4.6 mm × 150 mm). The elution solvent comprised a mixture of 30% water and 70% acetonitrile, with a set flow rate of 1.0 mL/min. Sample injections were standardized at a volume of 20 μL, and UV absorbance was monitored at a wavelength of 230 nm. The degradation rate (*DRt*) of CBs was quantified as the ratio of remaining CBs concentration post-reaction to its initial concentration, expressed in percentage terms. *DRt* was determined using the Equation 1:

$${DR}_{t}\;\left(\%\right)=\frac{C_{0}-C_{t}}{C_{0}}\times 100\%$$ (1)

where *C*_*0*_ represents the initial concentration of CBs prior to the reaction, and *C*_*t*_ denotes the concentration of CBs at time *t*.

Degradation intermediates were characterized employing a SCIONSQ-456-GC (Bruker Co., Ltd.) triple quadrupole mass spectrometry system, interfaced with an Agilent RTX-5 capillary column (30 m × 0.25 mm i. d., 0.25 μm). Nitrogen served as the carrier gas, maintaining a flow rate of 1.5 mL/min. The injector was heated to 280°C. The thermal program initiated at 60°C, sustained for 3 min, followed by a 10°C/min increment to reach 150°C, where it was maintained for 0.5 min. The temperature was then elevated to 180°C at a rate of 5°C/min and held for 2 min, subsequently increased to 280°C at 20°C/min, and finally stabilized at 280°C for 2 min. The injection was performed with a split ratio of 100:1.

Chloride ions produced in the reaction system were analyzed by ICS (Thermo Scientific Dionex ICS 5000+). The eluent used for IC analysis was KOH/methanesulfonic (isocratic elution 15 mM/20 mM). The anion/cation suppressor was used, and the pump flow rate was 1 and 0.36 mL/min.

### 2.6 Estrogenic activity study

To evaluate the estrogenic potential of CB solutions before and after treatment, estrogen-sensitive MCF-7 human breast cancer cells were employed. These cells were detached with trypsin and then resuspended in phenol red-free DMEM, supplemented with 5% charcoal/dextran-treated Fetal Bovine Serum (FBS) and 1% penicillin/streptomycin. This was conducted over a period of 72 h, with media replenishment occurring every 24 h. The cultures were maintained in a humidified environment with 5% CO_2_ at a constant temperature of 37°C. Post-incubation, the cells were harvested via centrifugation, then seeded into a 96-well plate at a density of 3 × 10^4^ cells/well. They were allowed to adhere within a 37°C incubator for an additional 24 h. Treatment regimens for the MCF-7 cells included untreated CB, enzyme-treated CB, or β-estradiol (E2) over 24, 48, and 72-h periods. Untreated MCF-7 cells served as the baseline control for cellular proliferation. At predetermined intervals, cells were collected for enumeration and stained with CCK8 to ascertain cell viability.

### 2.7 Molecular docking

The three-dimensional (3D) conformation of *Trametes versicolor* laccase, as denoted by the Protein Data Bank (PDB) code 1GYC, was retrieved from the PDB repository (http://www.rcsb.org/pdb/home/home.do). Concurrently, the 3D structures of CB and its isomers, 1, 2-DCB (code: 7239), 1, 3-DCB (code: 10943), and 1, 4-DCB (code: 4685), were sourced from the PubChem database (http://pubchem.ncbi.nlm.nih.gov/). Molecular docking of CB and its DCB isomers into the enzymatic active sites of laccase was performed using Autodock, employing the Lamarckian genetic algorithm for optimization. The parameters for docking were adapted from those delineated by Behbahani et al. (2016), with certain modifications to suit this specific analysis. A minimum of 20 independent docking runs were conducted, generating up to 10 potential binding poses per run. Subsequently, the most favorable conformation, determined based on binding affinity and stability, was selected for an in-depth interactional profile analysis utilizing LigPlot^+^.

### 2.8 Molecular dynamics simulations

Molecular Dynamics (MD) simulations of protein-ligand complex were performed utilizing the GROMACS 2020.3 computational platform (Wu et al., 2023). The AMBER99SB-ILDN force field and the General Amber Force Field (GAFF) were employed to define the parameter and topology for proteins and ligands, respectively. The dimensions of the simulation box were configured to ensure that the distance between any protein atom and the boundary of the box was greater than 1.0 nm. The simulation box was filled with explicit solvent using the Simple Point Charge (SPC216) water model, and the water molecules were replaced with Na⁺ and Cl⁻ counterions to achieve electrical neutrality in the simulation system. Energy minimization was performed using the steepest descent algorithm for 5.0 × 10^4^ steps to minimize the overall energy of the system and reduce any unrealistic contacts or atomic overlaps. Following energy minimization, the temperature of system was first stabilized at 300 K under the canonical ensemble (NVT) for 100 ps to reach the first phase equilibrium. Subsequently, an isothermal-isobaric ensemble (NPT) simulation at standard atmospheric pressure was conducted for 100 ps to achieve the second phase equilibrium. The principal aim of these simulations was to refine the interplay among the target protein, solvent, and ions to achieve a thoroughly pre-equilibrated simulation environment. Subsequently, unrestrained position simulations for all systems were executed at 300 K and 1 bar pressure within the NPT ensemble over a duration of 30 ns Temperature and pressure regulation were facilitated through the V-rescale and Parrinello-Rahman algorithms, respectively (Kawata and Nagashima, 2001), with coupling time constants set at 0.1 and 0.5 ps The computation of Van der Waals interactions was carried out employing the Lennard-Jones potential. All bond lengths within the atoms were constrained using the LINCS algorithm. Long-range electrostatic interactions were computed using the Particle Mesh Ewald (PME) method, with a Fourier spacing of 0.16 nm. The binding free energies were calculated using the gmx_mmpbsa. The visual display, analyze, and animation of trajectories were achieved with the visual molecular dynamics (VMD) software 1.9.3 version and PyMOL 2.4.1 version (Carretero-González et al., 2005).

### 2.9 Statistical analysis

Data are reported as the average derived from three independent measurements. The determination of statistical significance was conducted through one-way analysis of variance (ANOVA), utilizing SPSS Statistics 25.0 software (IBM Inc., Armonk, NY, United States). A P-value less than 0.05 was indicative of statistically significant differences.

## 3 Results and discussion

### 3.1 Laccase-mediated degradation of CB

**FIGURE 1:**
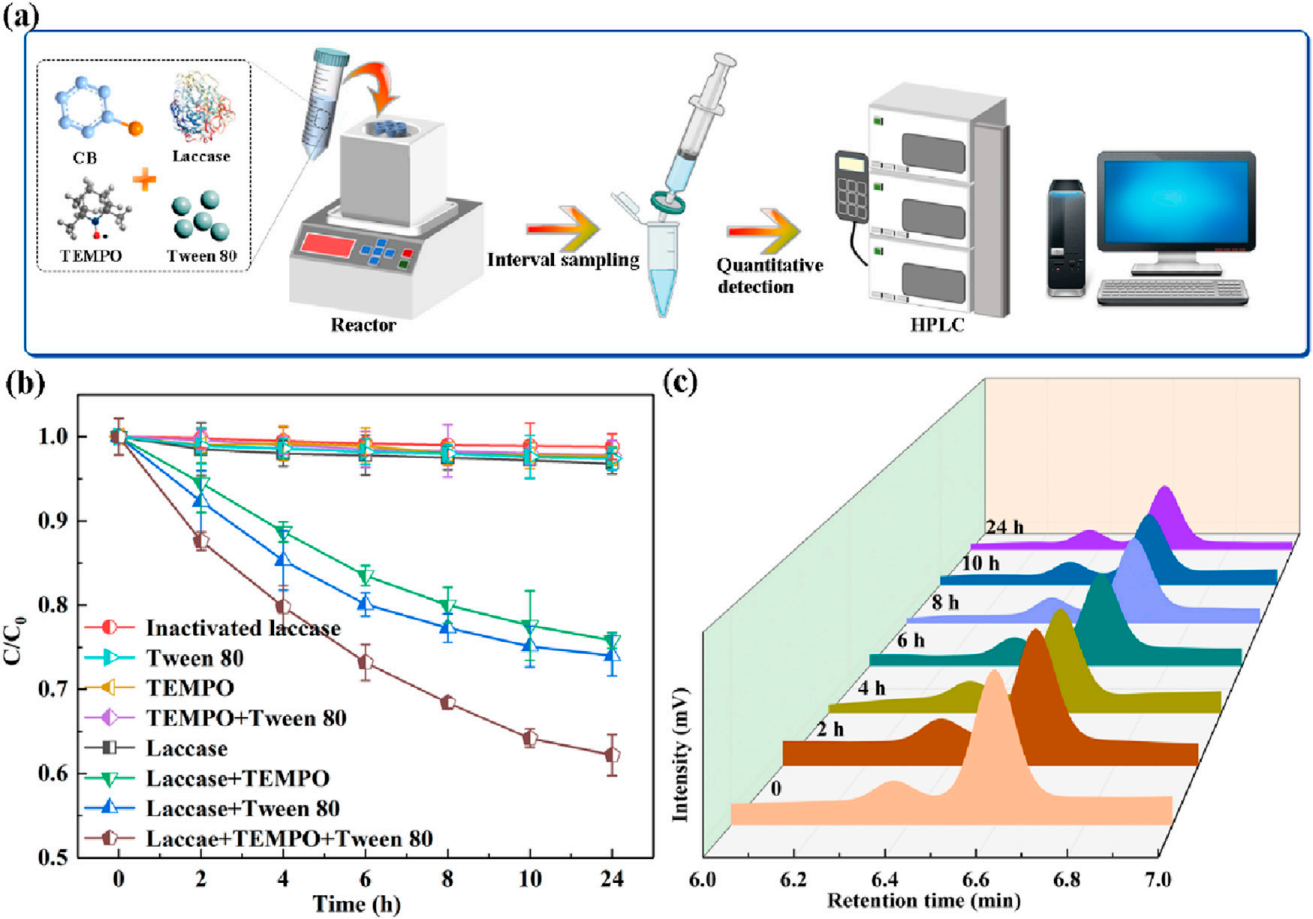
Schematic illustration of the experimental using laccase-assisted system to degrade CB **(A)**. Degradation efficiency of CB in different systems **(B)**. HPLC chromatograms of CB degradation by SALM system at different reaction times **(C)**.

The degradation of CB (initial concentration 25 mg/L) in an aqueous solution was investigated using both a laccase system and a Surfactant-Assisted Laccase-Mediator (SALM) system. The experimental setup is illustrated in Figure 1A. The concentration of CB during the degradation process was monitored via HPLC, which determined a retention time of 6.567 min for CB (Supplementary Figure S1A). A standard curve was established by linear regression of the HPLC peak area against known CB concentrations, enabling the accurate quantification of CB content at various time points (Supplementary Figure S1B). As depicted in Figure 1B, the laccase system alone demonstrated a limited CB degradation rate of approximately 3%, which is attributed to the low redox potential of enzyme. This observation underscores the necessity of incorporating a redox mediator to facilitate the degradation of non-phenolic substrates (Paul et al., 2024). In this context, three typical redox mediators HBT, ABTS, and TEMPO, were evaluated as potential mediators for CB degradation by laccase. The TEMPO demonstrated superior degradability compared to other mediators in the laccase-mediated system, as delineated in Supplementary Figure S2. The CB degradation efficiency enhanced by 21.41% upon the addition of TEMPO compared to the system employing only laccase. To further enhance the degradation rate, the addition of Tween 80, a surfactant known to improve bioremediation by increasing the solubility and mobility of hydrophobic organic compounds (Lyu et al., 2020), was explored in conjunction with the laccase-TEMPO system. The combined laccase-TEMPO system with Tween 80 (SALM system) exhibited a significant improvement in CB degradation, achieving 42.2% degradation after 24 h of incubation, which is 17.79% higher than the laccase-TEMPO system (Figure 1C), thereby highlighting the enhanced catalytic potency of the SALM system. Control experiments, involving the independent addition of TEMPO, Tween 80, or their combination, did not result in any discernible CB degradation, affirming that laccase is the critical component within the SALM system.

### 3.2 Optimization of reaction parameters and the stability of laccase

**FIGURE 2:**
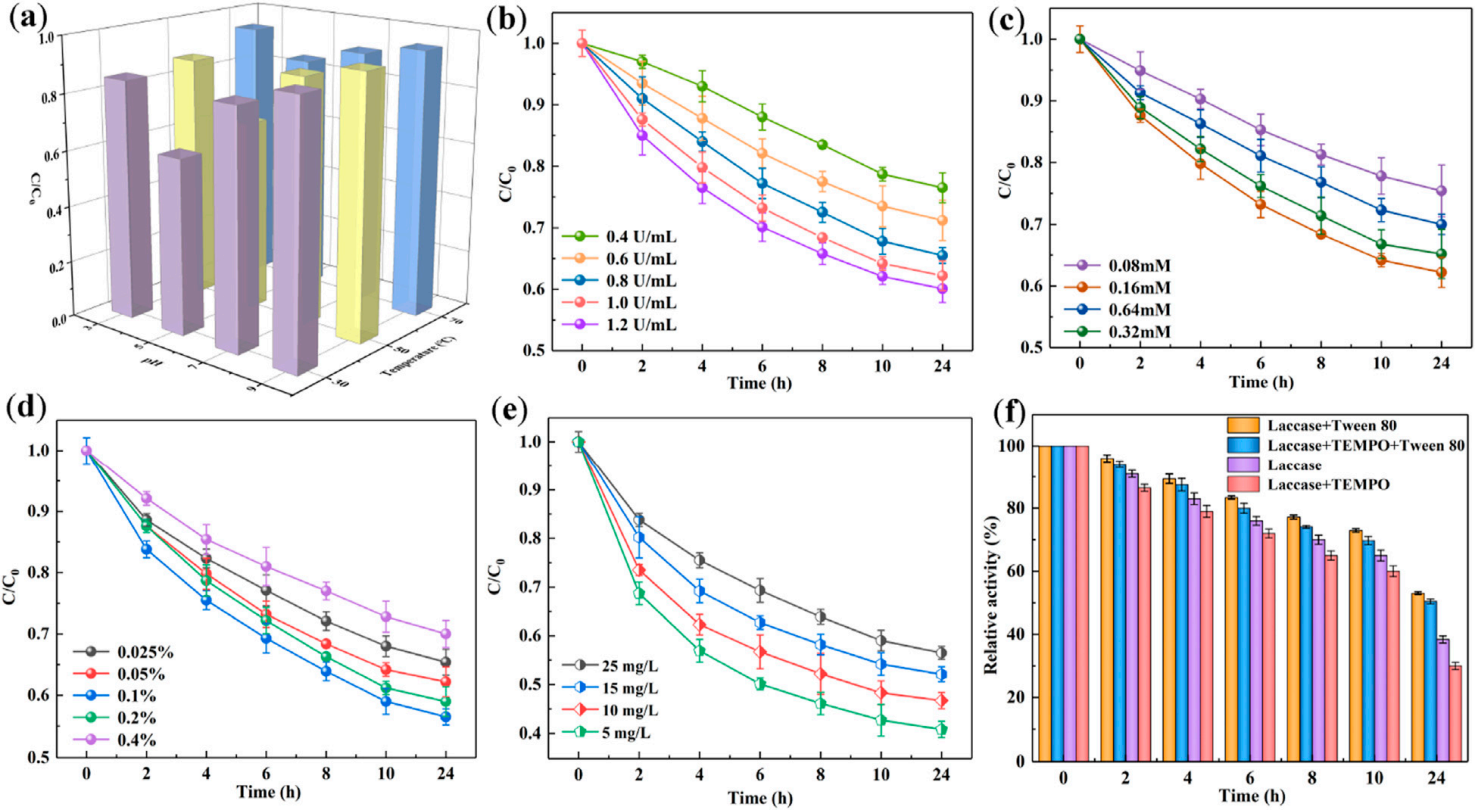
The effect of pH values and temperatures **(A)**, concentrations of laccase **(B)**, TEMPO **(C)**, Tween 80 **(D)** and CB **(E)** on CB degradation using SALM system. Stability of laccase in different reaction mediums **(F)**.

A systematic investigation was conducted to evaluate the impact of initial reaction parameters on the CB degradation rate by the SALM system. The effects of pH and temperature on CB degradation were examined across a pH range of 3.0–7.0 and temperatures of 30, 50, and 70°C. As depicted in Figure 2A, elevating the temperature from 30°C to 70°C markedly impeded the biocatalytic degradation efficacy of CB. This reduction can be attributed to heat-induced conformational alterations in the laccase, leading to thermal denaturation. Consequently, at elevated temperatures, the oxidizing capacity of laccase was adversely impacted, diminishing the biodegradation efficiency. Furthermore, the pH of the solution was also found to significantly influence the degradation rate of CB. Degradation experiments were extended over a broader pH spectrum, ranging from 3.0 to 9.0 (Figure 2A). The data revealed that the degradation rate of CB peaked at pH 5.0 (30°C), and subsequently declined with increasing pH levels. This trend correlates with the optimal catalytic activity and stability of laccase observed at pH 5.0 (Supplementary Figure S3). Extremes in pH, whether strongly acidic or weakly basic, could detrimentally impact the structural integrity of the amino acid chains in laccase molecules, thereby disrupting their functional conformation. Based on these findings, subsequent CB degradation experiments will be conducted at an optimized temperature of 30°C and a pH of 5.0, conditions that maximize both the catalytic activity and stability of the laccase.

Figure 2B delineates the correlation between the initial dosage of laccase and the degradation efficiency of CB in the system augmented with TEMPO and Tween 80. The laccase concentration was varied within the range of 0.4–1.0 U/mL. Within this range, an increase in laccase dosage corresponded to a higher conversion rate of CB. Specifically, a laccase concentration of 1.0 U/mL resulted in a 42.2% degradation of CB, compared to a 25% degradation observed at a concentration of 0.4 U/mL.However, when the laccase activity was further increased from 1.0 to 1.2 U/mL, the improvement in degradation efficiency was marginal, with an enhancement of less than 5%. This observation suggests the presence of a saturation point, beyond which further increases in laccase concentration yield diminishing returns in degradation efficiency. Thus, a laccase concentration of 1.0 U/mL was determined to be optimal for the degradation of CB in the reaction system.

Given that CB is not a natural substrate for laccase, the synthetic mediator TEMPO was employed to enhance the oxidation potential of laccase. Figure 2C illustrates the relationship between TEMPO concentration and the conversion rate of CB. The investigation revealed that a progressive enhancement in CB degradation efficiency was correlated with the gradual increase of TEMPO from 0.08 to 0.16 mM, culminating in maximum efficacy at a TEMPO concentration of 0.16 mM. Subsequently, a diminishing trend in degradation efficiency was noted as the TEMPO concentration was further increased from 0.16 to 0.64 mM. These findings indicate that TEMPO exerts a facilitative effect on the degradation of CB, with an optimal concentration determined to be 0.16 mM for achieving maximum degradation efficiency.

Surfactants present a viable option for enhancing the solubilization of organic contaminants. In this context, the widely utilized non-ionic surfactant, Tween 80, was assessed for its efficacy in facilitating the degradation of CB by the laccase-TEMPO system. The influence of varying concentrations of Tween 80 on CB degradation was systematically investigated across five different levels (0.025%, 0.05%, 0.1%, 0.2%, and 0.4%). As depicted in Figure 2D, an optimal Tween 80 concentration of 0.1% was identified, corresponding to a maximum CB degradation rate of 43.5%. This suggests that an incremental increase in surfactant concentration (0.025%–0.1%) proportionally enhanced the solubility of CB, thereby facilitating its degradation. However, concentrations of Tween 80 significantly exceeding the critical micelle concentration (CMC) led to the formation of extensive micelles. This restricted degradation by reducing the apparent solubility of the pollutant, as the excessive micelles encapsulated CB, thereby impeding its accessibility to the laccase-TEMPO system (Prak and Pritchard, 2002). Consequently, a further escalation in the concentration of Tween 80 (0.2% and 0.4%) resulted in an increased partitioning of CB molecules into the micelles. This sequestration effect significantly impeded the interaction between CB and laccase, as the molecules were encapsulated within the micellar structures. Hence, this led to a notable reduction in the overall degradation rate, highlighting the critical balance between surfactant concentration and pollutant accessibility in the degradation process.

Moreover, Figure 2E elucidates the impact of varying initial concentrations of CB on its biodegradation efficiency. A negative correlation was observed, whereby the degradation efficiency of CB diminished progressively as the initial concentration in the solution was increased from 6.25 to 50 mg/L. Specifically, after 24 h of treatment, the degradation efficiency was recorded at 56.2% for a 6.25 mg/L CB solution, in contrast to 35.7% for a 50 mg/L solution. This trend can be attributed to the fixed concentration of laccase and TEMPO used in the experiments, which in turn generated a specific quantity of oxoammonium cations. These cations, which are crucial for the oxidation process, might not suffice to effectively degrade higher concentrations of CB. Consequently, the degradation efficiency is likely to diminish as the CB concentration surpasses the stoichiometric capacity of the active species generated, underscoring the importance of optimizing reactant concentrations for efficient pollutant degradation.

The applicability of laccase in various industrial scenarios is critically dependent on its stability. To assess this, an investigation was conducted to examine the changes in laccase enzymatic activity after incubation at an optimal pH of 5.0 and temperature of 30°C for 24 h, in the presence of the chemicals essential for CB degradation, namely, TEMPO and Tween 80. Figure 2F illustrates that laccase exhibited instability in the presence of TEMPO, with a marked decrease in its activity, potentially attributable to the detrimental impact of intermediate free radicals generated during the reaction. Conversely, the integration of Tween 80 into the laccase-TEMPO system notably ameliorated the relative activity of laccase to 50.43% over 24 h. This enhancement implies that Tween 80 mitigates the adverse effects of TEMPO and imparts a stabilizing influence on laccase. A plausible explanation for this phenomenon might be the capacity of surfactants to modulate the microenvironment surrounding laccase, particularly affecting hydrophobic amino acid residues such as Tyrosine and Tryptophan. This modulation likely leads to a less polar surrounding or a confinement within the laccase structure, thereby fostering favorable folding and stabilization of the enzyme (Chan et al., 2024).

### 3.3 Degradation mechanism of CB by SALM system

The generation of chloride ion (Cl^−^) with respect to time during SALM treatment of CB solution was shown in Supplementary Figure S4. The initial CB concentration was established at 25 mg/L, and theoretically, 7.89 mg/L of Cl^−^ would be expected as a result of complete dechlorination. Following a 24-h treatment period, the concentration of Cl^−^ detected amounted to 3.278 mg/L, corresponding to a dechlorination efficiency of 41.55%. The majority of the residual chlorine was associated with the unreacted CB. Additionally, it is plausible that some chlorine might have been incorporated into various chlorinated intermediate compounds formed during the CB degradation process.

**FIGURE 3:**
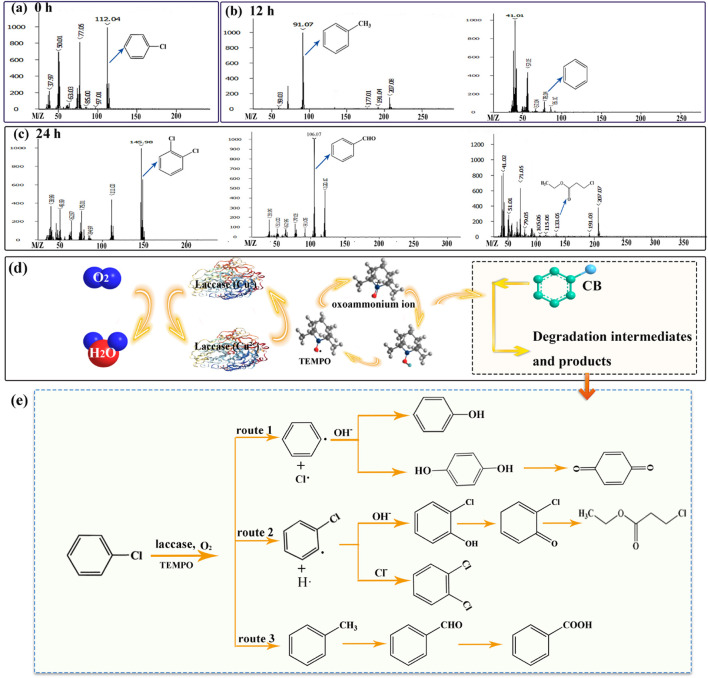
GC-MS profiles of CB **(A)**, degradation intermediates **(B)** and products **(C)**. Schematic illustration **(D)** and the hypothetical degradation route **(E)** for degrading CB via the SALM system.

In the course of CB degradation, various intermediates and products were identified through GC-MS. The spectrum of identified compounds encompassed aromatic compounds, benzene and its derivatives, nitrogenous organics, and chlorinated aliphatics, among others. Notably, the predominant intermediates and products included toluene (m/z = 91.07), benzene (m/z = 78.04), o-dichlorobenzene (m/z = 146), benzaldehyde (m/z = 106.07), and ethyl 3-chloropropionate (m/z = 133.05). The mass spectra corresponding to these compounds are depicted in Figures 3B, C. The emergence of nitrogenous organics was anticipated, given the introduction of TEMPO into the system, which is known to generate nitrogen oxide radicals. These radicals are likely to engage in subsequent interactions with aromatic rings, leading to the formation of nitrogen-containing organic compounds.

The schematic illustration of SALM system for CB degradation was shown in Figure 3D. TEMPO was oxidized by laccase during the single electron transfer to form oxoammonium ion, which then acted as the actual oxidizing agent for conversion and degradation of CB. Through the identification and analysis of the degradation intermediates and products, we speculated the degradation pathway of CB, as depicted in Figure 3E. In route 1, phenyl radical was formed by SALM catalyzed CB in the presence of O_2_, and its subsequent attacked by OH^−^ presented in the solution, resulting in the formation of phenol, or hydroquinone to further generate *p*-benzoquinone. In various studies, benzoquinone has been reported as one intermediate in the degradation of CB (Jose and Philip, 2019; Mtibaà et al., 2020; Wang et al., 2018). The C_6_H_4_Cl· formed through route 2 could convert to 2-chlorophenols and 1, 2-dichlorobenzene by reaction with OH^−^ and Cl^−^ presented in the solution, and 2-chlorophenols might undergo further oxidation to form 2-chlorobenzoquinone and subsequent degradation. Toluene would be formed by the reaction of CH_3_ groups liberated from any of the ring-opening products with benzene (route 3), it might be further degraded through the formation of benzaldehyde and benzoic acid. In this study, the possible pathway of SALM system degrading CB was speculated through the partially identified metabolites. And the specific degradation pathway will be demonstrated and analyzed in the next work.

### 3.4 Estrogenic activity of laccase treated CB solution

The measurement of estrogenic activity was determined through the proliferation rate of cells exhibiting responsiveness to EDCs stimulation. The human breast cancer cell line, MCF-7, which epitomizes estrogen receptor-positive cells, was employed as a model for estrogen-dependent cell proliferation. The E-screen assay, employing the MCF-7 cell line, is widely acknowledged as a sensitive and reliable method for detecting the estrogenic activity of environmental pollutants (Elfadul et al., 2021). In this study, both untreated and bioremediated CB solutions were examined. Concurrently, a solution (1 × 10^−7^ M) of 17 β-estradiol (E2) was employed as a positive control to benchmark estrogenic activity. Moreover, the pure culture medium was also subjected to the estrogenic activity test, serving as a negative control.

**FIGURE 4:**
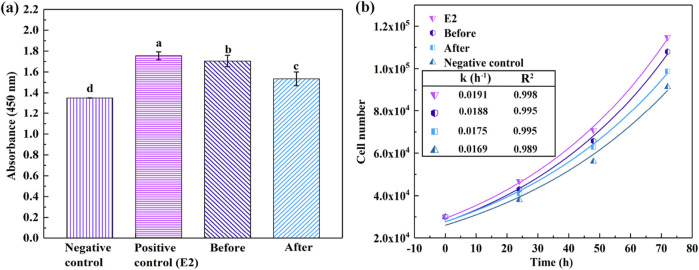
Quantification of MCF-7 cells after 72 h of cultivation under varied conditions **(A)**, alongside growth trajectories of MCF-7 cells over time **(B)**. Distinct alphabets in **(A)** signify statistically significant disparities (P < 0.05).

Figure 4A depicts the proliferation of MCF-7 cells over a 72-h culture period under various stimuli. The data indicated that the untreated CB solution instigated cell proliferation akin to that elicited by E2, which is recognized as a full agonist. Unlike partial agonists, which induce significantly lower cell proliferation compared to E2, full agonists demonstrate considerable affinity and intrinsic activity. Conversely, the proliferation induced by the laccase-treated CB solution was less pronounced than that of the untreated counterpart, implying that the biodegradation of CB was concurrent with a reduction in its estrogenic activity. The residual estrogenic activity observed in the laccase-treated CB solution might be attributed to the remaining CB molecules and the synergistic interactions among the compounds persisting in the solution. In Figure 4B, cell proliferation is represented as a function of growth time, adhering to the model *N(t)* = *N*_*0*_ *e(kt)*, where *N(t)* represents the cell count at time “*t*” under different stimulation cultures, and the constant “*k*” is proportionally related to the rate of growth as compared to the baseline cellular population. These findings substantiate the hypothesis that laccase can attenuate the estrogenic activity of endocrine disruptors, thereby facilitating the detoxification process (Husain and Qayyum, 2013; Varga et al., 2019).

### 3.5 Influence of structure of CBs on degradation performance

**FIGURE 5:**
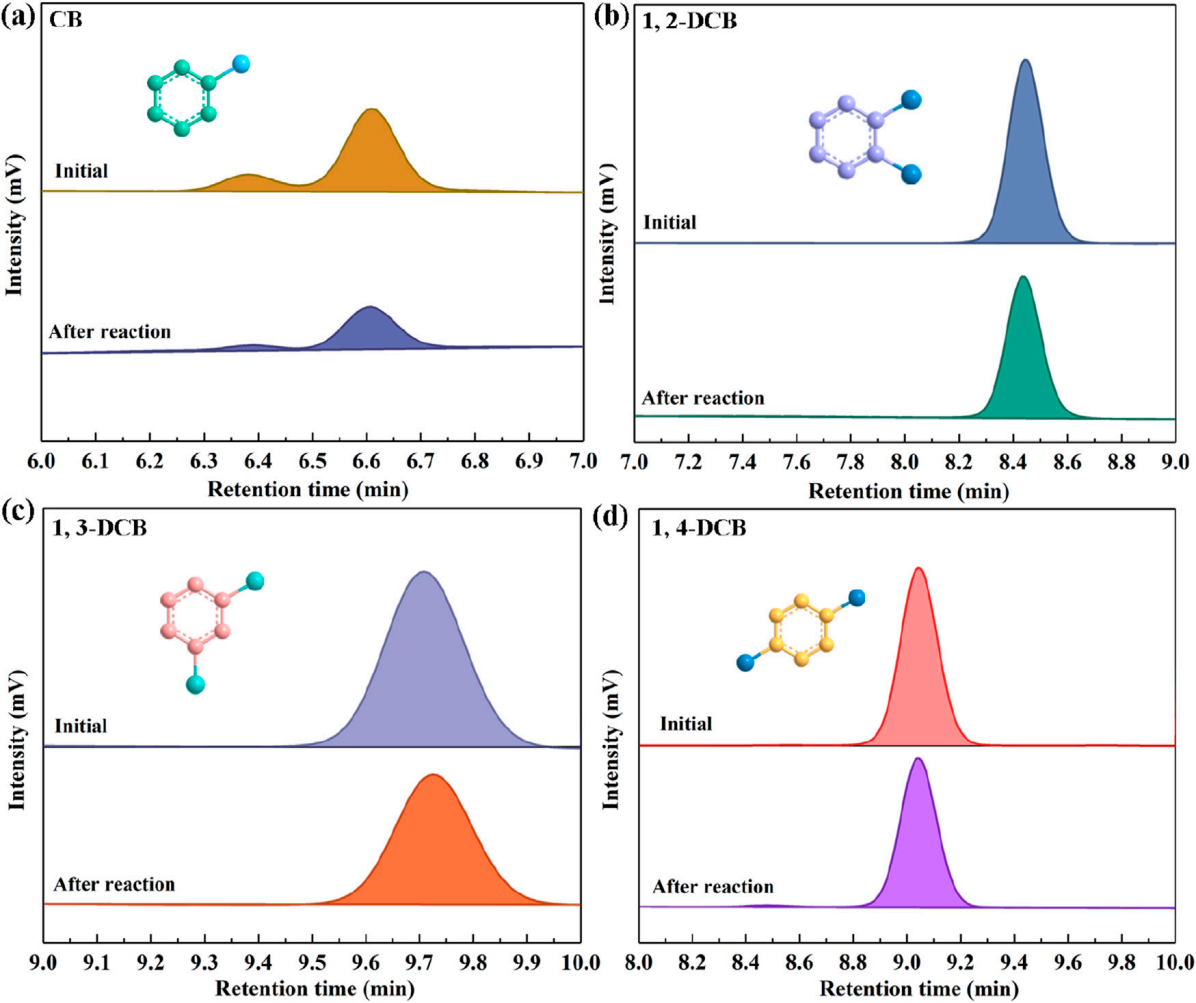
HPLC chromatograms of CB **(A)**, 1, 2-DCB **(B)**, 1, 3-DCB **(C)** and 1, 4-DCB **(D)** before and after the degradation reaction. Reaction conditions: initial concentration, 25 mg/L; temperature, 30°C; pH, 5.0; laccase, 1.0 U/mL; TEMPO, 0.16 mM; Tween 80, 0.1%.

In this study, the SALM system was employed to systematically study the degradation efficacy of CB and its isomers 1,2-DCB, 1,3-DCB, and 1,4-DCB, as illustrated in Figure 5. HPLC analysis was employed to ascertain the presence of the substrates CB, 1,2-DCB, 1,3-DCB, and 1,4-DCB at retention times of 6.654, 8.433, 9.75, and 9.1 min, respectively. Quantitative analysis of the degradation process involved calculating the residual concentrations of the four CBs at specific time intervals, based on HPLC peak areas. The degradation rates, as presented in Table 1, followed the order of CB (43.5%) > 1,3-DCB (28.56%) > 1,2-DCB (24.42%) > 1,4-DCB (17.6%). The observed variations in degradation rates can be attributed to distinct phenomena. Firstly, the increase in the number of chlorine atoms within the molecule resulted in a decrease in the enzyme-catalyzed degradation efficacy, as evident in the comparison between CB and DCB. This finding suggests that the presence of chlorine atoms limits the degradation of CBs by the SALM system. Furthermore, the position of chlorine atom substitution also exerted an influence on degradation, as demonstrated by the comparison between 1,2-DCB, 1,3-DCB, and 1,4-DCB. Specifically, it was more challenging to convert DCB with para-chlorine substitution, indicating that the SALM system exhibited lower efficiency in degrading ortho- and meta-chlorine substituted compounds. The impact of chlorine atoms on CBs degradation can be attributed to a combined effect of σ-electron induced effect, π-electron conjugation effect, and steric hindrance effect (Lei et al., 2021). This comprehensive analysis provides valuable insights into the factors influencing the enzymatic degradation of CB and its isomers by the SALM system.

**TABLE 1:** The residual concentration and degradation rate of CB and DCB isomers after degradation by SALM system.

Chlorobenzenes	Residual concentration (mg/L)	Degradation rate (%, 24 h)
CB	14.125	43.5^a^
1, 2-DCB	18.895	24.42^b^
1, 3-DCB	17.86	28.56^b^
1, 4-DCB	20.60	17.6^c^

^a^ Distinct alphabetic indicators denote a statistically significant difference (P < 0.05).

### 3.6 Analysis of docking results

**FIGURE 6:**
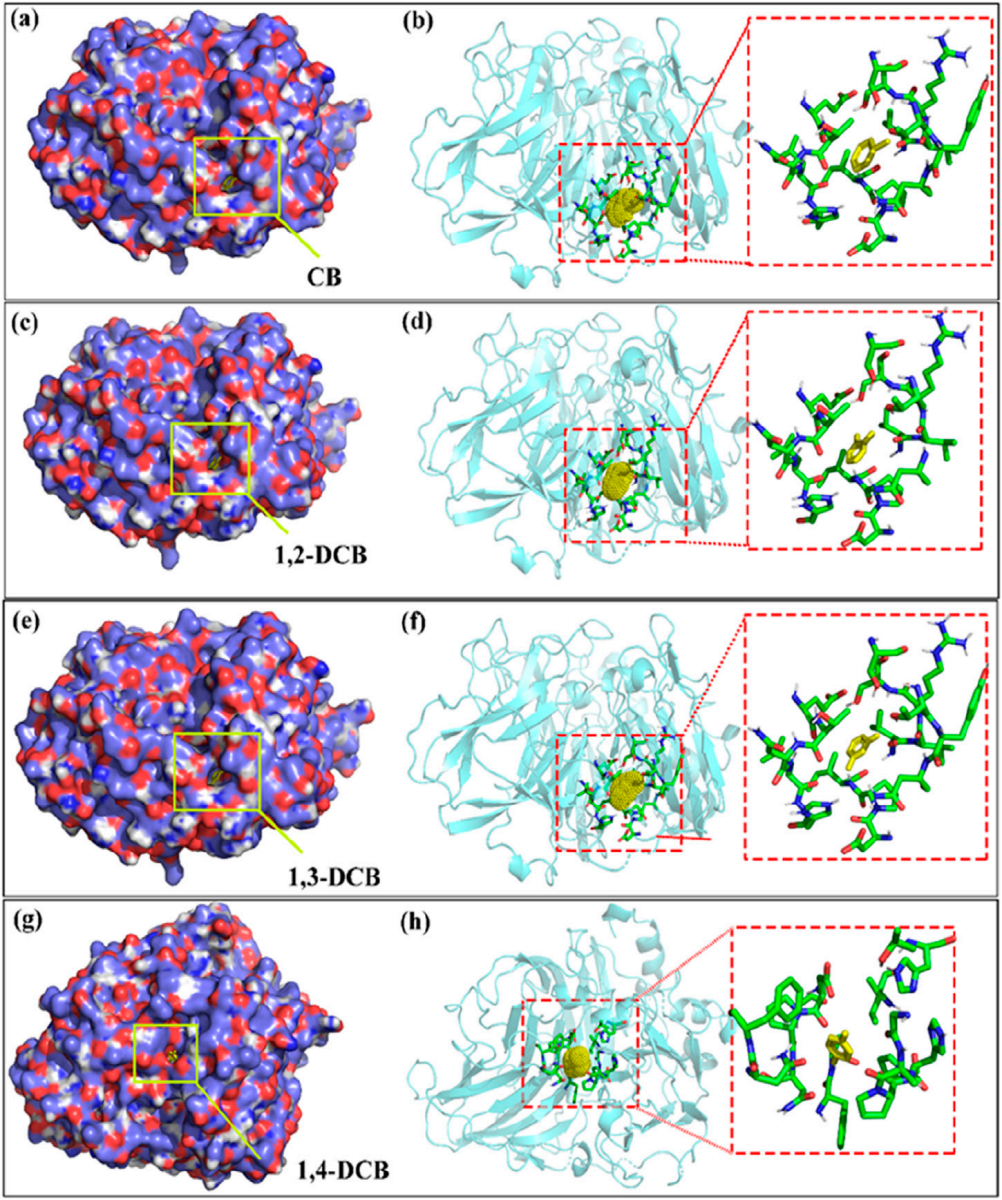
Binding orientations, detailed views of CB and DCB isomers in laccase [**(A, B)** laccase-CB; **(C, D)** laccase-1, 2-DCB; **(E, F)** laccase-1, 3-DCB; **(G, H)** laccase-1, 4-DCB] (In Binding orientations, carbon and oxygen are represented as blue and red spheres, respectively. In detailed views, CBs or DCB isomers shown in yellow stick models, the sticks around them are shown as the amino acid residues within 6 Å).

The results of the docking study between laccase and CB and its isomers, presented in Figure 6 and Table 2, elucidated the binding modes and poses within the cavities of the isoform complex (Figures 6A, C, E, G). The laccase-ligand binding images affirmed the integration of CB, 1,2-DCB, 1,3-DCB, and 1,4-DCB into the laccase structure. Figures 6B, D, F, H showcased detailed perspectives of CB and DCB isomers in laccase binding sites, highlighting diverse ligand binding patterns. The revealed results suggested varying ligand binding sites on the laccase structure. Noteworthy is the similarity in docking sites among CB, 1,2-DCB, and 1,3-DCB, indicating a shared interaction pattern with laccase. These findings enhance the understanding of molecular interactions between laccase and CB derivatives, offering valuable insights for potential applications in enzymatic processes.

**TABLE 2:** Amino acid residues involved in hydrophobic contacts, profile of binding affinity for the best docking complex of laccase and ligands.

Enzyme-ligand complex^a^	Amino acid residues involved in hydrophobic contacts	Binding energy (kJ mol^−1^)
Laccase-CB	Ile 421 Leu 305 Val 406 Pro 420 Asn 416 Ser 409 Thr 303 Glu 302	−21.56
Laccase-1, 2-DCB	Ile 421 Leu 305 Val 406 Pro 420 Asn 416 Ser 409 Thr 303 Glu 302	−20.76
Laccase-1, 3-DCB	Ile 421 Leu 305 Val 406 Pro 420 Ser 409 Thr 303 Glu 302	−21.47
Laccase-1, 4-DCB	Phe 265 Pro 394 Asn 206 Ile 455 Ala 393 Gly 392 Pro 207 Asn 204 Asn 208	−19.13

^a^ Refers to the best docking complex.

Hydrophobic contacts and hydrogen bonds within the optimal pocket were investigated using LigPlot^+^. The varying efficiencies observed in laccase-mediated degradation of four CB homologues could be attributed, in part, to distinct binding interaction mechanisms. Notably, no hydrogen bonds were identified between laccase and CB homologues, underscoring the significant role played by hydrophobic contacts, given the inherent hydrophobic nature of CB homologues. Amino acid residues implicated in hydrophobic contacts were identified as key contributors to these interactions. Examining the amino acid residues involved in hydrophobic interactions (Table 2), CB and DCB isomers with chlorine atoms in meta- and ortho-positions exhibited analogous hydrophobic contacts, while DCB with a para-chlorine atom displayed differences. Common residues, including Ile 421, Leu 305, Val 406, Pro 420, Asn 416, Ser 409, Thr 303, and Glu 302, played pivotal roles in forming hydrophobic bonds essential for laccase-CB and laccase-1,2-DCB (ortho-position) complexes. In the case of laccase-1,3-DCB (meta-position), the amino acid residues involved in hydrophobic interactions were essentially identical to the aforementioned complexes, except for the absence of Asn 416. Conversely, for the laccase-1,4-DCB (para-position) complex, common residues participating in hydrophobic contacts included Phe 265, Pro 394, Asn 206, Ile 455, Ala 393, Gly 392, Pro 207, Asn 204, and Asn 208. The structural features and binding orientations of CB and DCB isomers were found to correlate with hydrophobic functionality. Complexes with similar ligand structures exhibited resemblances in binding orientations, sharing more common residues involved in hydrophobic effects. Figure 6 illustrated that the binding complexes, laccase-CB, laccase-1,2-DCB, and laccase-1,3-DCB, displayed similar docking sites and binding orientations, whereas laccase-1,4-DCB differed. Consequently, the amino acid residues participating in hydrophobic contacts in laccase-1,4-DCB were distinct from the other complexes.

The binding energy data for the enzyme-ligand complexes are presented in Table 2. The binding energies for CB, 1, 2-DCB at the ortho-position, 1, 3-DCB at the meta-position, and 1, 4-DCB at the para-position were calculated as −21.56, −20.76, −21.47, and −19.13 kJ mol^−1^, respectively. The negative sign of the binding energy indicates the spontaneity of the laccase-mediated substrate degradation. Specifically, a smaller binding energy between enzymes and substrates implies a greater affinity, enhancing the likelihood of pollutant degradation due to stronger interactions (Hongyan et al., 2019). It was observed that ligand structures with fewer substituents tended to exhibit higher binding affinities, as evidenced by a comparison of the binding energies between CB and DCB. This trend further highlighted that, within the complexes of DCB isomers binding with laccase, the binding affinities followed the order of 1,4-DCB (para-position) < 1,2-DCB (ortho-position) < 1,3-DCB (meta-position). The reduced binding affinity observed for DCB with para-chlorine atoms was influenced by steric hindrance. Intriguingly, DCB isomers with ortho- and meta-chlorine atoms exhibited minimal differences in binding affinity, orientation, and hydrophobic effects, indicating analogous patterns and mechanisms in their interactions. These docking results align with the experimental findings on the degradation of CBs isomers by the SALM system, where the degradation rate of 1, 4-DCB was lower than that of 1, 2-DCB and 1, 3-DCB. The observed binding affinity may be intricately linked to the efficiency of enzyme-ligand interactions, providing insights into the tightness of the binding complex (Zhang et al., 2012). The in-depth understanding of the enzyme-ligand interactions facilitates the development of CB homologues biodegradation technologies, and provides a theoretical basis for the bioremediation of such pollutant.

### 3.7 Analysis of MD results

In the enzymatic hydrolysis of pollutants, MD technology serves as a valuable tool to computationally simulate the trajectory and interaction dynamics between enzyme and pollutant molecules. Through numerical calculations, MD simulations can unveil the intricate details of the reaction mechanism between enzymes and pollutants (Awasthi et al., 2015). In this section, MD simulation was employed to scrutinize the conformational changes within the laccase-CB complex in both the binary laccase-CB system (LC1) and the tertiary laccase-TEMPO-CB system (LC2) over a 30 ns simulation period. An exhaustive analysis of RMSD, Rg, SASA, RMSF, hydrogen bonding, and MM-PBSA parameters enabled the comprehensive evaluation of the stability, interaction dynamics, and binding energy of the complex throughout the binding process. This multifaceted approach aims to provide deeper insights into the reaction mechanism governing laccase-mediated degradation of CB.

**FIGURE 7:**
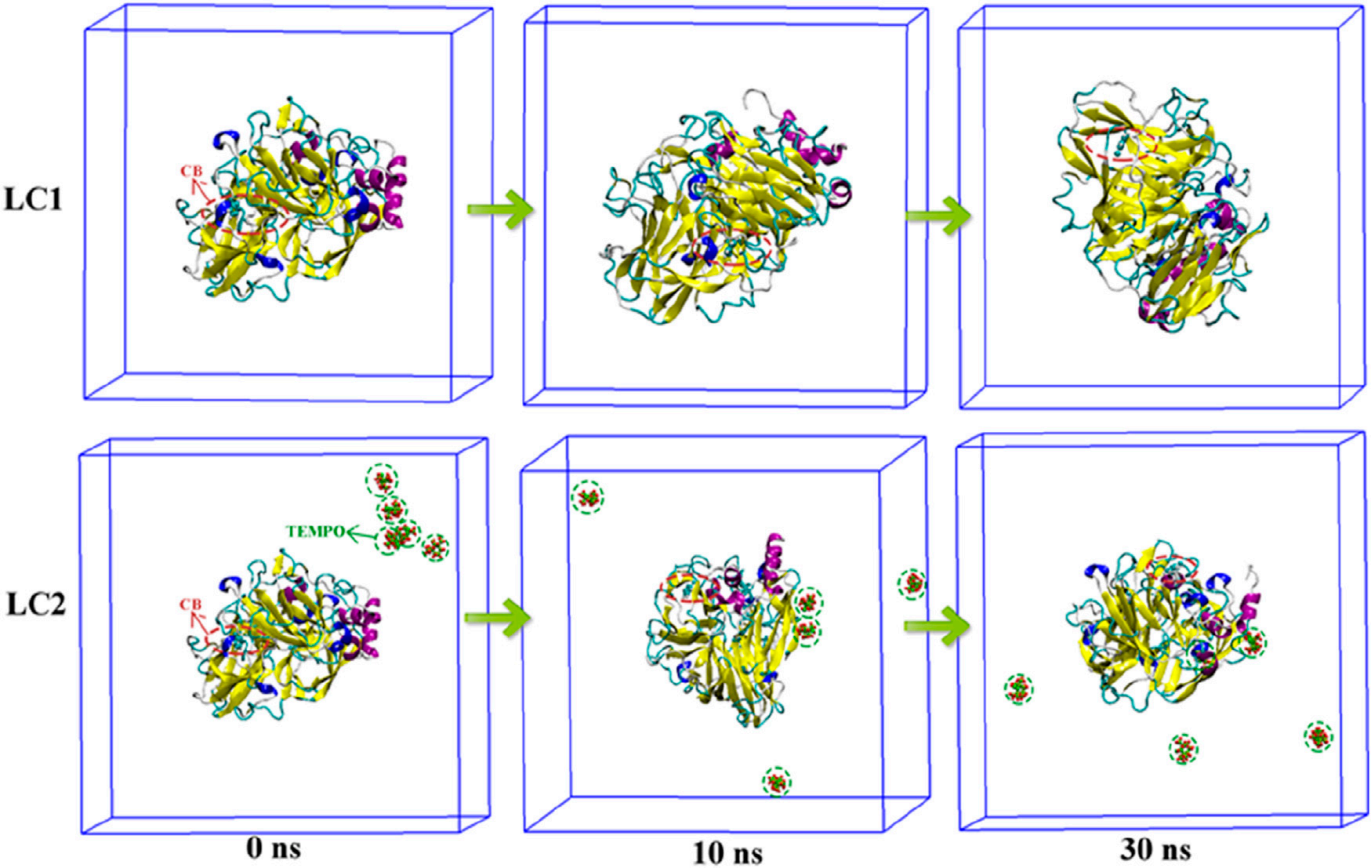
Conformational transformation of complexes in LC1 and LC2. The laccase protein molecule is depicted in a Cartoon representation, the CB ligand molecule is highlighted within a red dotted oval box, and the TEMPO mediator molecule is enclosed by a green dotted line oval box.

The conformational dynamics of LC1 and LC2 during MD simulation are illustrated in Figure 7. CB in LC1 permeated the interior of the laccase at 0 ns, and the laccase conformation underwent continuous changes throughout the simulation. Remarkably, the binding position of CB within laccase remained stable. Upon the introduction of the TEMPO molecule in LC2, the TEMPO molecule gradually approached laccase and penetrated the laccase molecule during the simulation. Additionally, CB molecules entered the laccase interior at 0 ns, and the binding site of CB to laccase exhibited consistency throughout the simulation duration. These findings underscore the mutual attraction and inherent affinity between CB molecules and laccase, suggesting a robust and stable interaction.

**FIGURE 8:**
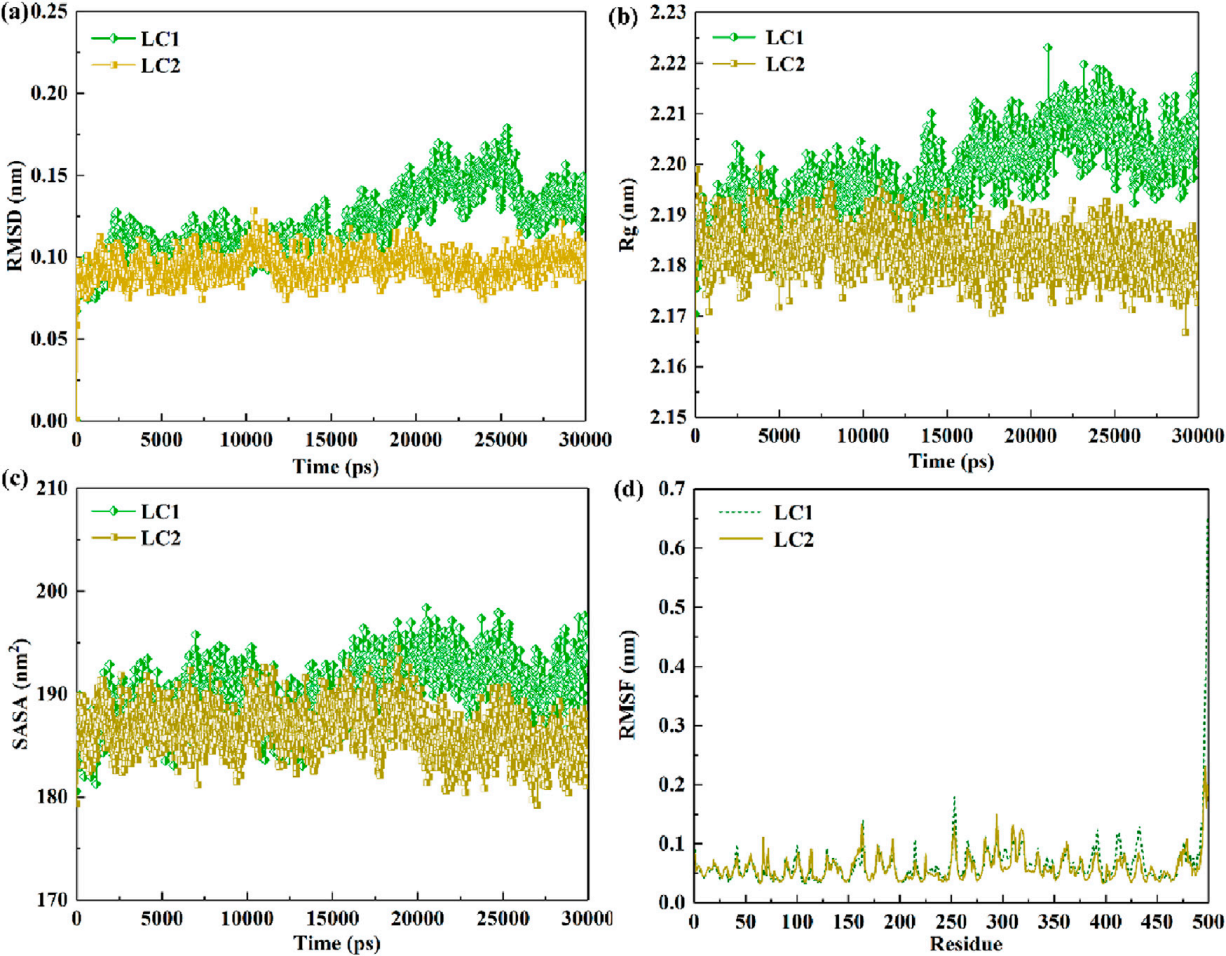
Dynamic evolution of RMSD values **(A)** and Rg values **(B)** for LC1 and LC2 over simulation time; SASA variations in laccase molecules **(C)** and RMSF fluctuations of laccase residues **(D)** within LC1 and LC2 throughout the simulation duration.

Within MD, RMSD serves as a pivotal gauge for quantifying dissimilarities between molecular structures. In the enzymatic hydrolysis of pollutants, RMSD becomes a crucial metric, reflecting the stability and structural variations in enzyme-pollutant binding at distinct time points (Hongyan et al., 2019). A smaller RMSD fluctuation and value denote heightened stability in enzyme-pollutant binding, indicative of minimal structural alterations throughout the binding process. Conversely, a larger RMSD fluctuation and value suggest diminished stability in enzyme-pollutant binding, signifying significant structural variations. Figure 8A illustrates the RMSD changes of laccase in both LC1 and LC2 over the 30 ns (30,000 ps) simulation period. This analysis provides a comprehensive view of the stability and dynamic structural shifts in enzyme-pollutant binding interactions during the simulation.

As depicted in Figure 8A, the RMSD value of laccase in LC2 remained relatively stable throughout the entire simulation, suggesting minimal structural changes in laccase during the CB binding process. In contrast, the RMSD value of laccase in LC1 exhibited stability up to 15,000 ps, followed by significant fluctuations from 17,500 to 27,500 ps, indicating structural alterations during this period. The mean RMSD values for LC1 and LC2 were (0.119 ± 0.020) nm and (0.094 ± 0.008) nm, respectively. Notably, the lower mean RMSD value of laccase in LC2 compared to LC1 indicates higher complex stability in LC2, highlighting a more consistent conformation of laccase in LC2.

Moreover, the Rg value is commonly utilized to assess structural changes and compactness of the enzyme during the binding of pollutants, serving as an indicator of binding stability between the enzyme and substrate. As observed in the Figure 8B, the Rg value of LC2 exhibited minimal fluctuations throughout the entire simulation, suggesting a relatively stable complex. In contrast, the Rg value of laccase in LC1 increased during the simulation, with significant fluctuations occurring from 17,500 to 27,500 ps, indicating a gradual loosening of the laccase structure within the LC1 system during CB binding. The average Rg values for LC1 and LC2 complexes were (2.198 ± 0.008) nm and (2.183 ± 0.004) nm, respectively. These values further underscore the superior stability of the complex in LC2 compared to LC1.

SASA proves insightful for evaluating the enzyme interaction with contaminants and changes in the exposed surface area of the enzyme structure to the solvent. Figure 8C portrays a stable SASA value for laccase in both systems throughout the 30,000 ps trajectory, with marginal fluctuations. However, it is discernible that the SASA value of laccase in LC2 experiences a downward trend, while LC1 exhibits an upward trend during the simulation. This implies that the binding in LC2 is more securely established compared to that in LC1.

RMSF is a widely employed metric for gauging changes in the vibrational amplitude of individual atoms, providing insights into the stability of enzymes during the binding of contaminants (Takur et al., 2023). In this segment, the RMSF variations in LC1 and LC2 over a 30,000 ps simulation were monitored, and the outcomes are presented in Figure 8D. The figure highlights notable fluctuations in both LC1 and LC2 within regions encompassing residues 150–172, 246–262, 382–393, 398–422, and 424–440, with the most significant oscillations occurring at residue position 499. Notably, the RMSF value of LC1 at the corresponding position exceeded that of LC2. Furthermore, the average RMSF values for LC1 and LC2 were calculated as (0.065 ± 0.043) nm and (0.06 ± 0.024) nm, respectively. When the enzyme binds to the pollutant, a decrease in the RMSF value indicates a reduction in the vibrational amplitude of the enzyme structure region binding to the pollutant, signifying enhanced stability in that region. Conversely, an increase in the RMSF value suggests instability in the enzyme region bound to the pollutant. Therefore, the observed results indicate that the binding position in LC2 exhibits greater stability than that in LC1.

**FIGURE 9:**
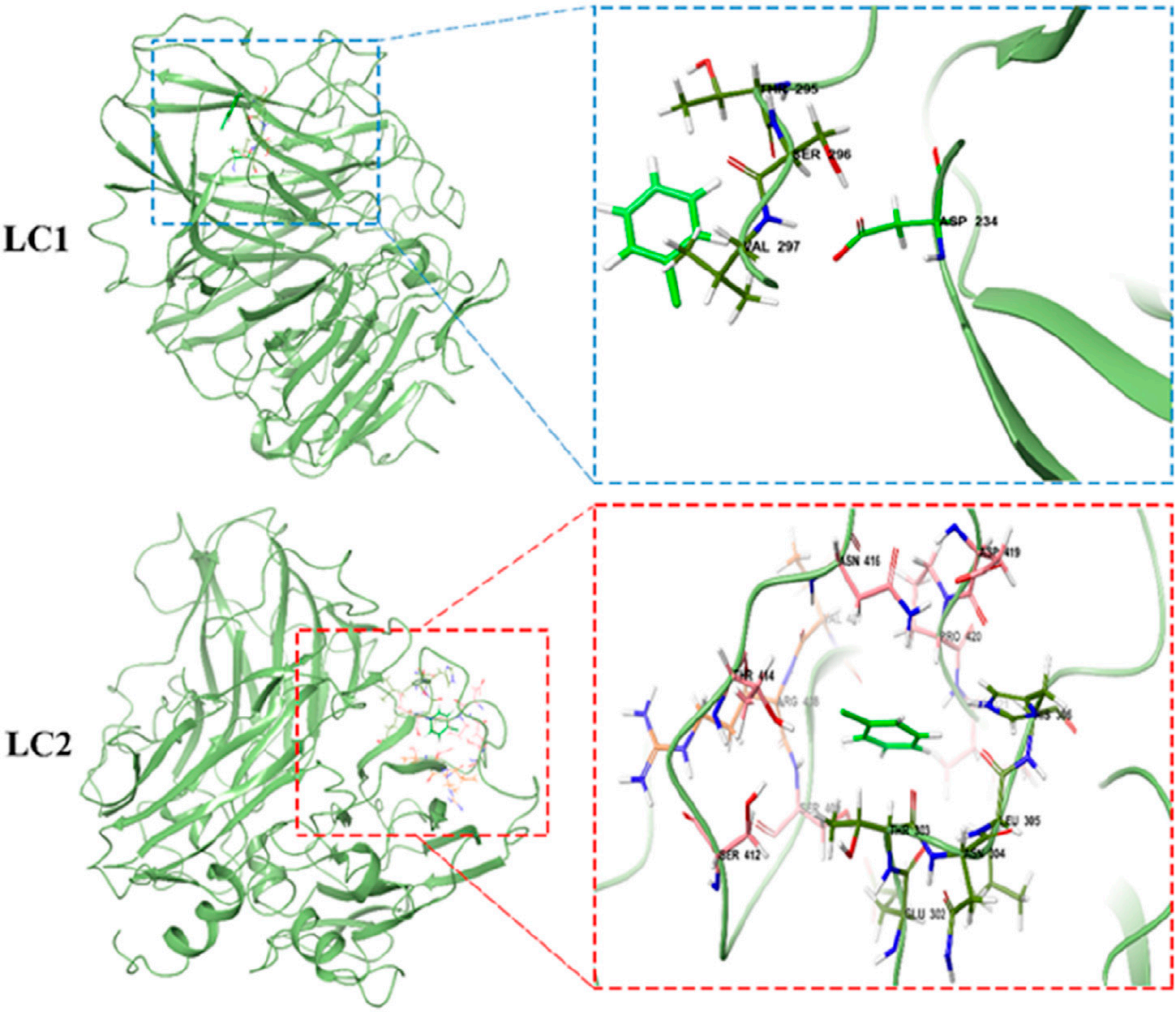
Detailed view of enzyme-substrate interactions for LC1 and LC2 at 30 ns simulation.

To comprehensively investigate the intricate interaction mechanism between laccase and CB within LC1 and LC2 systems, a detailed scrutiny of binding sites within the complexes was conducted through a 30 ns molecular dynamics simulation, as illustrated in Figure 9. Notably, the examination revealed an absence of hydrogen bonding between laccase and CB in both LC1 and LC2, highlighting that their interaction is predominantly governed by hydrophobic contacts.

In the LC1 system, four key amino acid residues namely Thr 295, Ser 296, Val 297, and Asp 234, were identified as the primary contributors to the interaction with CB. Conversely, in LC2, this interaction extended to approximately 14 amino acid residues, prominently featuring Asn 416, Pro 420, Thr 303, Leu 305, Arg 408, Ser 401, and His 306. These findings underscore the nuanced differences in the binding patterns between laccase and CB within the two systems. It is noteworthy that the independent binding affinity of laccase and CB was inherently feeble. Intriguingly, upon the introduction of TEMPO into the system, a substantial enhancement in the contact between CB and the active site of laccase was observed, resulting in a notable augmentation of their interaction and affinity.

**FIGURE 10:**
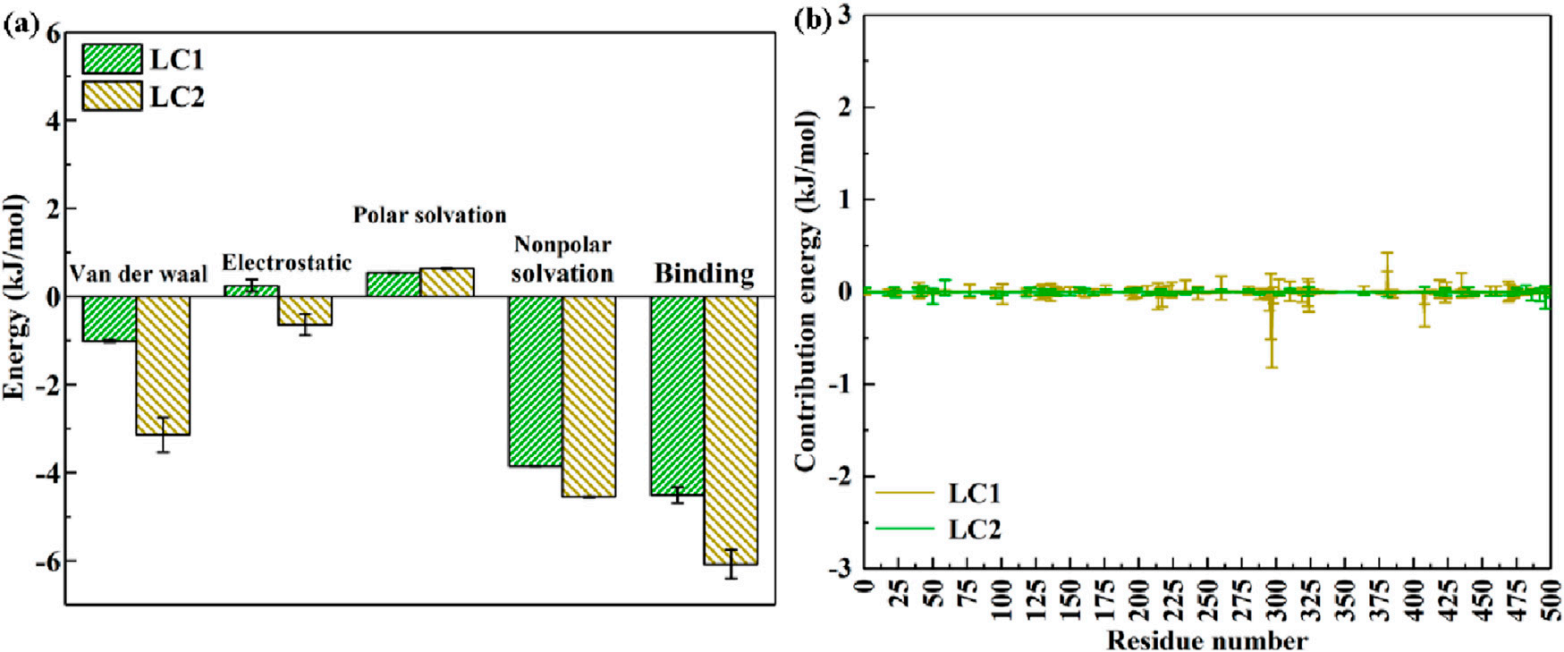
Energy contribution of complexs in LC1 and LC2 **(A)**; the contribution energy of each amino acid residue of laccase in LC1 and LC2 to binding energy **(B)**.

Following this, the interaction energy between laccase and CB within LC1 and LC2 was subjected to meticulous analysis utilizing the MM-PBSA approach. Figure 10 elucidates the distribution of average interaction energy during the final 5,000 ps of the molecular dynamics simulation within LC1 and LC2 systems. This analysis provides a deeper understanding of the energetics underlying the laccase-CB interaction, offering valuable insights into the stability and dynamics of the complexes in the specified time frame.

In Figure 10A, the binding energies of LC1 and LC2 are (−4.508 ± 0.181) kJ mol^−1^ and (−6.081 ± 0.329) kJ mol^−1^, respectively. Notably, both cases exhibit negative binding energies, indicative of the capability of enzyme for catalytic substrate degradation. Generally, a more negative binding energy implies a higher affinity between the enzyme and the substrate. Consequently, the outcomes reveal that laccase in LC1 and LC2 exhibits a discernible binding affinity with CB, with LC2 displaying a stronger affinity compared to LC1. Furthermore, the VDW energies of the LC1 and LC2 complexes are recorded as (−1.013 ± 0.025) kJ mol^−1^ and (−3.144 ± 0.389) kJ mol^−1^, respectively. VDW represents an interaction force between microscopic particles, determined by the calculation of charge distribution between particles (Bhatt and Maheshwari, 2022). The substantial VDW in LC2 indicates a robust interaction force, fostering evident attraction when particles are in proximity. Conversely, the smaller VDW in LC1 signifies a weaker interaction force between laccase and CB. The COU values, denoting the interaction between enzyme and pollutant, provide insights into their combination likelihood (Bhatt et al., 2023). A positive COU of LC1 [(0.251 ± 0.134) kJ mol^−1^] suggests a mutual repulsive force, implying limited proximity between laccase and CB. Conversely, the negative COU of laccase and CB in LC2 [(−0.638 ± 0.242) kJ mol^−1^] indicates TEMPO-induced charge alterations, creating a mutual attraction between laccase and CB.

Additionally, PSE and NPSE values evaluate interaction strength with solvents and predict substance solubility. PSE values for LC1 and LC2 are (0.54 ± 0.009) kJ mol^−1^ and (0.64 ± 0.022) kJ mol^−1^, respectively, reflecting weak interactions with polar solvents due to the low solubility of CB. Larger NPSE values in LC1 and LC2 suggest heightened interaction forces with non-polar solvents, indicating potentially higher solubility and easier dissolution. Moreover, employing the MM-PBSA method, further exploration of amino acid residues contributing significantly to laccase-catalyzed bioremediation of CB is presented in Figure 10B.

As depicted in Figure 10B, LC1 exhibits minimal participation of amino acid residues in the energy contribution to laccase binding with CB. In contrast, within LC2, the predominant amino acid residues contributing to energy include Ile 298, Ser 296, Ile 382, and Ser 409. Ile amino acids are pivotal constituents of enzyme molecules, providing crucial structural support and stabilizing the enzyme structure. Furthermore, Ile residues can engage with substrates, facilitating substrate recognition and binding, thereby promoting catalytic reactions (Holliday et al., 2009). Notably, Ser amino acids, being active centers of enzymes, play multifaceted roles. They can partake in substrate binding, conversion, and release during reaction processes. Additionally, Ser residues can serve as nucleophiles or base catalysts in enzyme-catalyzed reactions, actively participating in the chemical catalysis process (Ribeiro et al., 2020). Simultaneously, the polar groups of Ser contribute to the interaction between the enzyme and the reaction substrate, stabilizing the three-dimensional structure of enzyme. This interaction profoundly influences the catalytic efficiency and specificity of the enzyme.

In summary, amino acids emerge as pivotal contributors to enzyme catalysis, playing an indispensable role in enhancing catalytic efficiency and specificity. Their diverse functions, ranging from structural stabilization to active participation in catalytic reactions, underscore their significance in influencing enzyme behavior.

## 4 Conclusion

This study initially evaluated the degradation efficiency of CB by laccase, LMS, and SALM, followed by an examination of the effects of reaction conditions on enzymatic degradation efficiency, including structural changes in CB, analysis of degradation products, and a detailed exploration of the interaction mechanism between laccase and CB through MD simulations. Subsequently, the estrogenic activity of the substrate solutions before and after enzymatic treatment was analyzed. Key findings include: low degradation rate of CB by laccase alone (3% after 24 h), which significantly increased to 24.41% with TEMPO and further to 37.8% with the addition of Tween 80 in the SALM system. Optimal conditions for the SALM system were pH 5.0, 30°C, with laccase at 1.0 U/mL, mediator concentration at 0.16 mM, and Tween 80 at 0.1% (w/v), achieving a 43.5% degradation rate. Additionally, Tween 80 not only enhanced the solubility of hydrophobic pollutants but also stabilized laccase activity. Dechlorination rate of CB by SALM reached 41.55% after 24 h, with major intermediates and products identified, suggesting possible degradation pathways. Molecular analysis indicated stable laccase-CB complexes in both LC1 and LC2 systems, with no hydrogen bonding observed. Binding energy analyses revealed the catalytic potential of laccase towards CB, with stronger affinity in the LC2 system. E-Screen assays post-SALM treatment showed reduced estrogenic activity in CB solutions, indicating effective biodegradation and concurrent reduction in estrogenicity.

## Data Availability

The original contributions presented in the study are included in the article/Supplementary Material, further inquiries can be directed to the corresponding authors.
