# Supplementary material for: Biocatalytic degradation of environmental endocrine disruptor chlorobenzene via surfactant-optimized laccase-mediator system
Source: Front Bioeng Biotechnol. 2024 Oct 14;12:1469029. doi: 10.3389/fbioe.2024.1469029 (PMC11513312; doi:10.3389/fbioe.2024.1469029)
Supplement: Supplementary file 1 [file DataSheet1.docx]

**Supporting Information**

**Biocatalytic degradation of environmental endocrine disruptor chlorobenzene via surfactant-optimized laccase-mediator system**

Dan Wang^1, 2^*, Guifang Huang^1, 2^, Chunming Yu^1, 2^, Yawen Wang^1, 2^, Nawon Baek^3,4^ and Ruofei Zhu^1, 2^*

^1^*College of Textile & Clothing, Xinjiang University, Urumqi, China.*

^2^*Xinjiang Key Laboratory of Intelligent and Green Textile, Xinjiang University, Urumqi, China*

^3^*Department of Clothing and Textiles, Kyungpook National University, Daegu, Korea.*

^4^*Center for Beautiful Aging, Kyungpook National University, Daegu, Korea.*

*Corresponding authors:

E-mail: ffwd@xju.edu.cn (Dan Wang); ruofeizhu@xju.edu.cn (Ruofei Zhu)


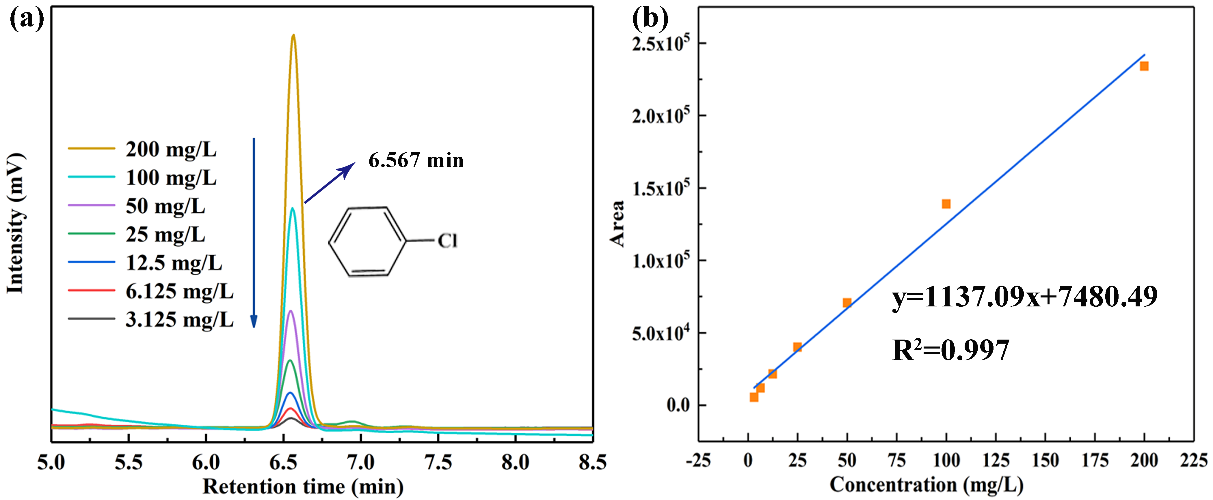


**Fig. S1.** HPLC chromatograms of standard CB solutions at a range of concentrations (a), and the standard curve of peak area as a function of CB concentration (b).


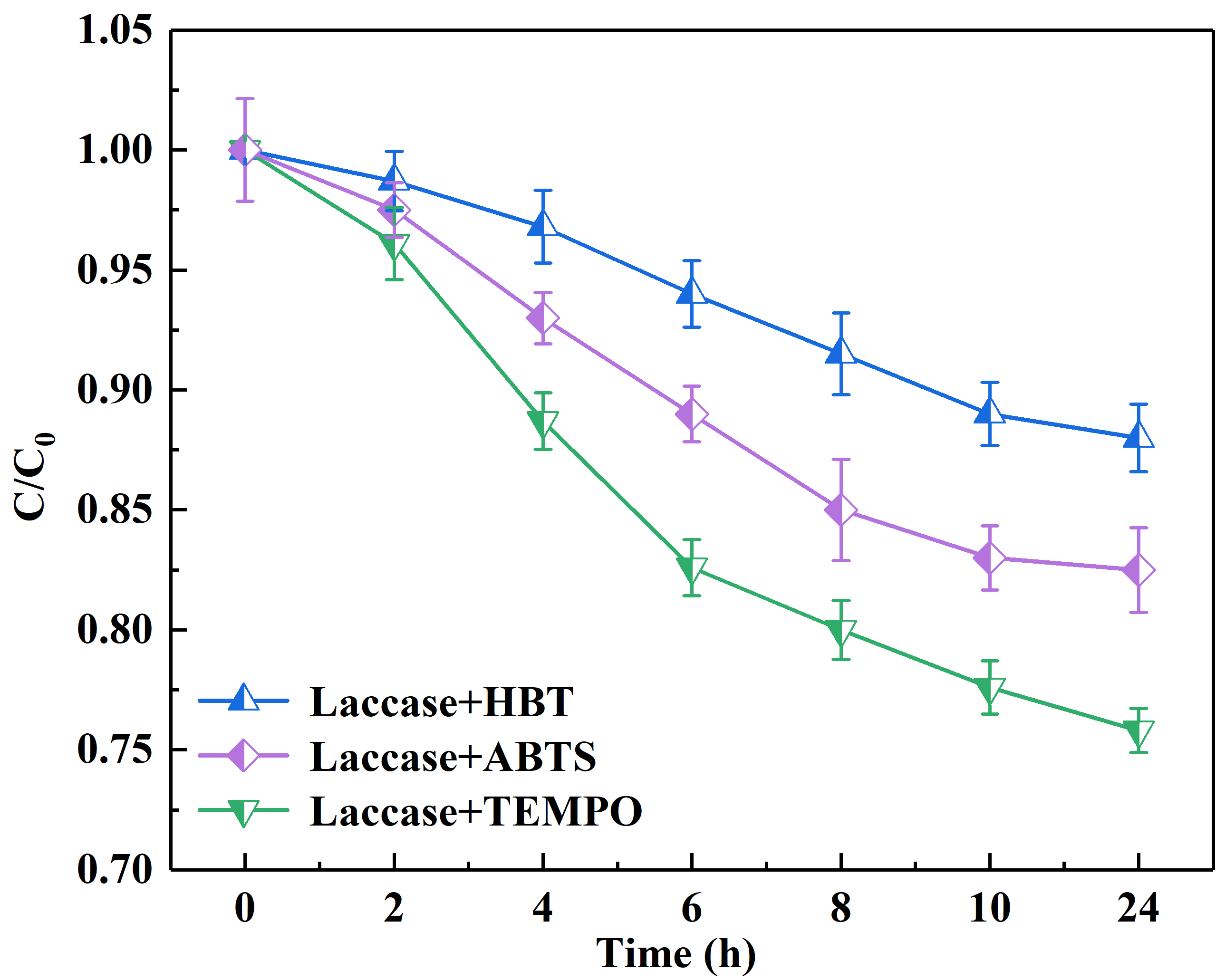


**Fig. S2.** Effect of HBT, ABTS, and TEMPO on the degradation of CB by laccase.


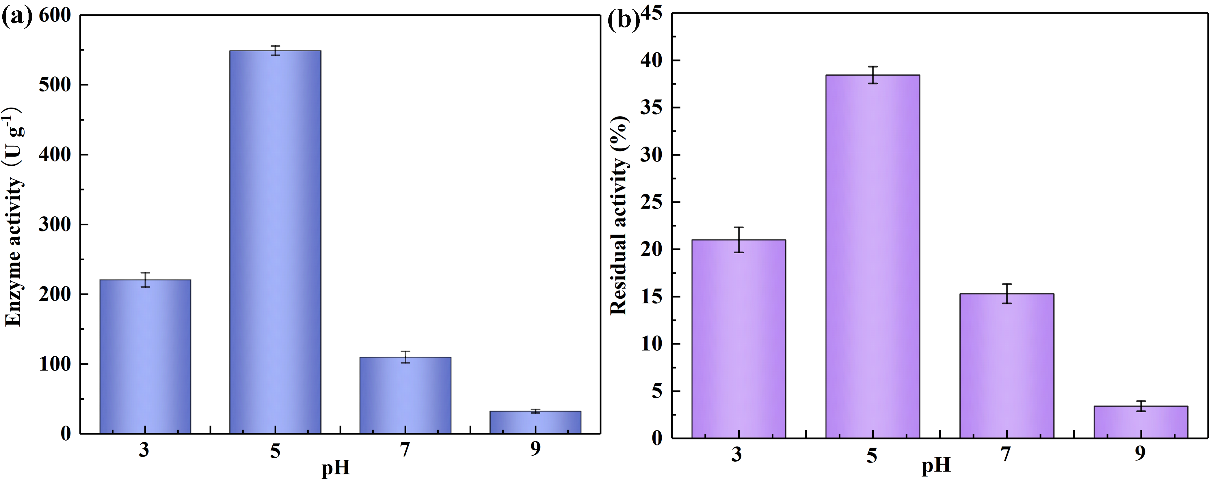


**Fig. S3.** Effect of pH on laccase activity (a) and residual activity of laccase after 24 h at different pH (b).


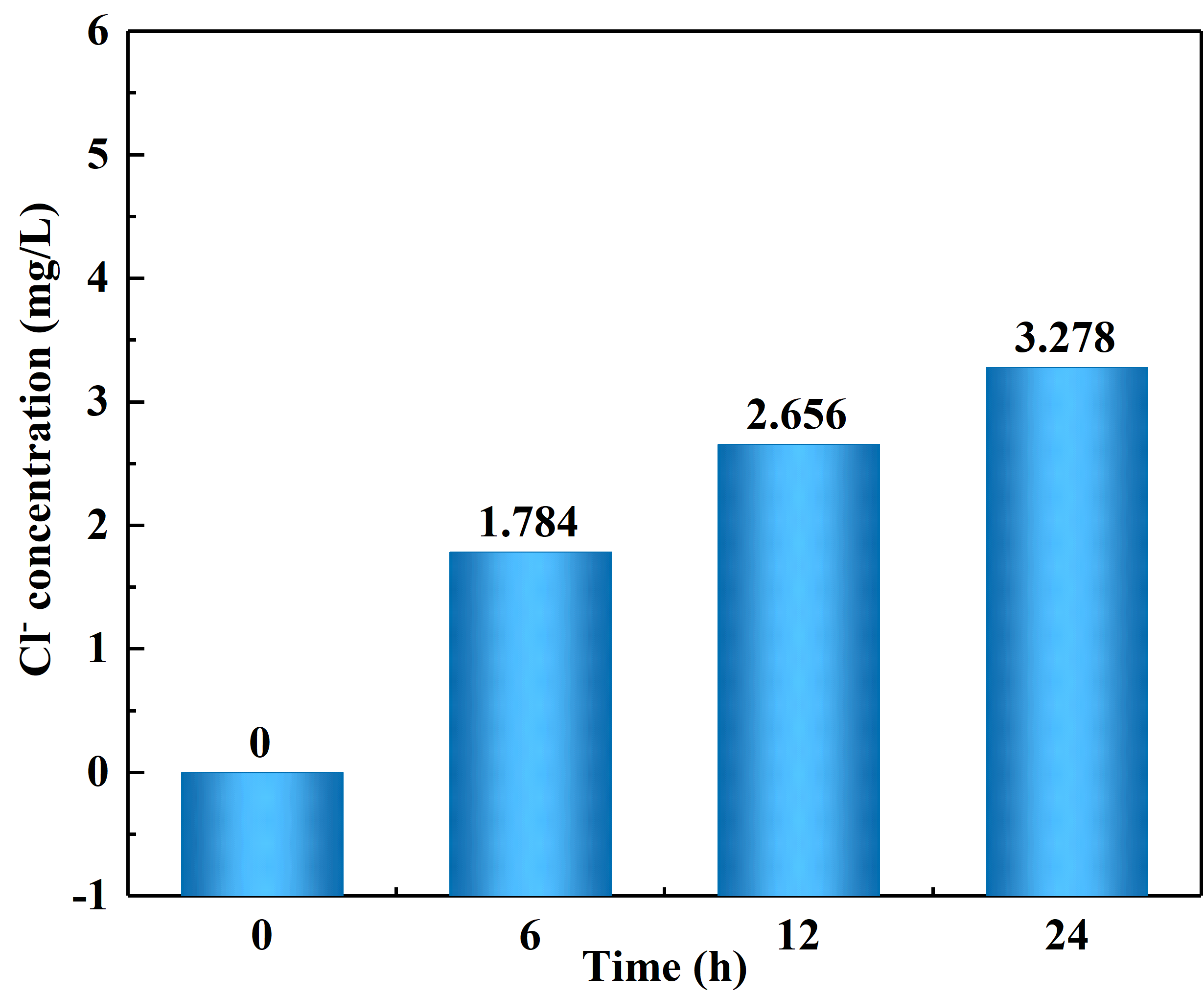


**Fig. S4.** The concentration of Cl^−^ generated from the dechlorinaton of CB in SALM system.
